# The Identification of DepB: An Enzyme Responsible for the Final Detoxification Step in the Deoxynivalenol Epimerization Pathway in *Devosia mutans* 17-2-E-8

**DOI:** 10.3389/fmicb.2018.01573

**Published:** 2018-07-17

**Authors:** Jason Carere, Yousef I. Hassan, Dion Lepp, Ting Zhou

**Affiliations:** Guelph Research and Development Centre, Agriculture and Agri-Food Canada, Guelph, ON, Canada

**Keywords:** deoxynivalenol (DON), reduction, 3-keto-DON, 3*-epi*-DON, detoxification, mycotoxin

## Abstract

Deoxynivalenol (DON) is one of the most common mycotoxins found in cereal grains and grains contaminated with DON can cause health issues for both humans and animals and result in severe economic losses. Currently there is no feasible method to remediate affected grains. The development of a biological method for detoxification is becoming increasingly more plausible with the discovery of microbes which can transform DON to a relatively non-toxic stereoisomer, 3-*epi*-DON. Although bacteria capable of detoxifying DON have been known for some time, it is only recently an enzyme responsible was identified. In *Devosia mutans* 17-2-E-8 (*Devosia* sp. 17-2-E-8) a two-step DON epimerization (Dep) pathway, designated as the Dep system, completes this reaction. DepA was recently identified as the enzyme responsible for the conversion of DON to 3-keto-DON, and in this report, DepB, a NADPH dependent dehydrogenase, is identified as the second and final step in the pathway. DepB readily catalyzes the reduction of 3-keto-DON to 3-*epi*-DON. DepB is shown to be moderately thermostable as it did not lose significant activity after a heat treatment at 55°C and it is amenable to lyophilization. DepB functions at a range of pH-values (5–9) and functions equally well in multiple common buffers. DepB is clearly a NADPH dependent enzyme as it utilizes it much more efficiently than NADH. The discovery of the final step in the Dep pathway may provide a means to finally mitigate the losses from DON contamination in cereal grains through an enzymatic detoxification system. The further development of this system will need to focus on the activity of the Dep enzymes under conditions mimicking industrially relevant conditions to test their functionality for use in areas such as corn milling, fuel ethanol fermentation or directly in animal feed.

## Introduction

Deoxynivalenol (DON, vomitoxin) is an economically important mycotoxin affecting cereals. It is produced by multiple fungal pathogens, such as *Fusarium graminearum*, during infection and disease development, and can act as a potent virulence factor (Proctor et al., 1995; Maier et al., 2006). *F. graminearum* costs the cereal industry millions in losses yearly, which can be amplified during years of high disease severity (McMullen et al., 1997), not only due to yield losses but also to grain contamination with mycotoxins, mainly DON. DON contamination is widespread throughout the globe (Streit et al., 2013) and can cause health issues for both humans and animals. When ingested by animals, DON leads to emesis and a loss of appetite (Forsyth et al., 1977; Rotter et al., 1996), while long term effects include reduced immunity, increased sensitivity to disease and a lack of weight gain (Bergsjø et al., 1993; Pestka et al., 2004). When grains contaminated with high levels of DON are processed, the resulting animal feed can have higher than acceptable limits; this results in discounts being applied to contaminated grains.

Reducing the amount of DON accumulation in grains is of high importance to industry and many strategies to mitigate the problem have been explored (Zhu et al., 2016). There are currently no effective chemical processes for effectively and economically detoxifying or eliminating DON during grain processing. DON has been shown to be detoxified by several bacterial species via specific modifications (Karlovsky, 2011). The de-epoxidation of DON has been demonstrated by several species and occurs under anaerobic conditions (Binder et al., 1997; Fuchs et al., 2002; Young et al., 2007). The C3 carbon is a common point of modification. Plants often glycosylate DON at the C3 position, but the resulting masked mycotoxin can be hydrolyzed back to DON via multiple mechanisms (Berthiller et al., 2011). The toxicity of DON can also be reduced by the acetylation of the C3 carbon (Kimura et al., 1998), but again the attached acetyl group can be hydrolyzed during digestion to reform DON. The oxidation and subsequent reduction of DON to produce 3-keto-DON and 3-*epi*-DON, respectively, has also shown to be a promising detoxification method. Both of these compounds have reduced toxicity compared to DON, especially 3-*epi*-DON (He et al., 2015a; Pierron et al., 2016). *Devosia mutans* 17-2-E-8 (*Devosia* sp. 17-2-E-8) has been shown to transform DON to 3-*epi*-DON through a two-step process (He et al., 2016; Hassan et al., 2017). Recently, the first enzyme in DON epimerization (Dep) pathway, DepA, was identified as a pyrroloquinoline quinone (PQQ)-dependent dehydrogenase (Carere et al., 2017). This enzyme readily converts DON to 3-keto-DON and can be expressed heterologously in *E. coli.*

**FIGURE 1:**
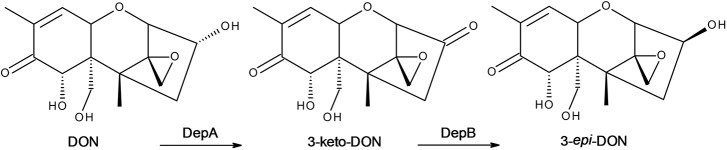
The DON epimerization pathway. DON is oxidized to 3-keto-DON by DepA followed by a reduction to 3-*epi*-DON by DepB.

Here we have identified DepB from *D. mutans* 17-2-E-8, the second enzyme in the DON detoxification pathway. The ability to convert DON to 3-*epi*-DON (**Figure 1**), a compound with at least 50-fold less toxicity, would be a large step forward in producing high quality feed even during large scale fusarium disease epidemics. Regular use of a treatment to detoxify DON would also help alleviate some of the long term issues/decreased productivity faced by livestock exposed even to low levels of DON. These enzymes could be used directly as a feed additive, incorporated into industrial feed production (either as pure enzymes or incorporated into microorganisms), or inserted into plants in an attempt to detoxify DON before harvesting.

## Experimental Procedures

### Chemicals

All chemicals were from Fisher Scientific (Nepean, ON, Canada) unless otherwise stated. DON was from Sigma–Aldrich (Oakville, ON, Canada) or TripleBond (Guelph, ON, Canada) and 3-keto-DON was from TripleBond. 3-*epi*-DON was previously purified and its identity confirmed (He et al., 2015b).

### Partial Purification of Potential DON Transforming Enzymes

A culture of *D. mutans* 17-2-E-8 was grown for 2 weeks at 28°C in LB medium supplemented with 34 μg ml^-1^ of kanamycin while shaking at 200 RPM. This starter culture was used to inoculate 4 L of LB medium which was incubated for 6 days at 28°C while shaking at 150 RPM. The culture was then centrifuged at 8,000 × *g* for 30 min to pellet the cells. The cell pellets were then frozen at -20°C.

Frozen pellets were thawed and cells resuspended in 50 mM Tris, pH 8.0 with 150 mM NaCl. The cells were then lysed by sonication using a QSONICA Q500 sonicator (Qsonica LLC., Newtown, CT, United States) while on ice. Sonication parameters were as follows: 30% amplitude, 30 s on and 50 s off for 20 cycles. The cells were allowed to cool for 10 min on ice before being subjected to another 20 cycles of sonication. The lysed cells were then centrifuged at 7,142 × *g* for 30 min to remove the insoluble fraction. The supernatant was filtered through a 0.45 μm syringe filter and Halt Protease Inhibitor Cocktail (Thermo Fisher, Mississauga, ON, Canada) without EDTA, was added.

The active fraction of the lysate was then salted out using ammonium sulfate precipitation (Duong-Ly and Gabelli, 2014). Briefly, the ammonium sulfate concentration was brought to 30% of saturation and insoluble proteins were removed by centrifugation at 7,142 × *g* for 20 min. The ammonium sulfate concentration was brought to 50% of saturation and the insoluble fraction was collected by centrifugation at 7,142 × *g* for 20 min. This was repeated for 70% saturation. Each of the above fractions was subjected to dialysis using Slide-A-Lyzer mini dialysis device 3.5K MWCO (Thermo Fisher), to remove the excess salt, and tested for the ability to convert 3-keto-DON to 3-*epi*-DON (DepB activity). The active fraction was then subjected to a heat treatment at 60°C for 15 min. The insoluble protein was removed by centrifugation at 7,142 × *g*. During this initial purification, enzymatic activity was assayed by adding 20 μL of protein solution to a solution with a final concentration of 100 μg ml^-1^ 3-keto-DON in Tris pH 8.0. The amount of 3-*epi*-DON produced was measured after approximately an hour and/or overnight.

The dialyzed active fraction obtained after ammonium sulfate precipitation and heat treatment, was subjected to anion exchange column chromatography using a HiTrap Q FF column (GE Healthcare) attached to AKTAPrime plus and monitored at 280 nm. The sample was loaded onto the column which had been equilibrated with 20 mM Tris (Buffer A). The column was then rinsed with 8 ml of Buffer A and proteins were eluted by a linear gradient from 0% Buffer B (20 mM Tris, 1 M NaCl) to 100% over 40 ml. Fractions containing the highest activity were pooled, concentrated by ultrafiltration using an Amicon Stirred Cell with an Ultracel 10 kDa ultrafiltration Disk (Millipore). The sample was then loaded onto a HiPrep 16/60 Sephacryl S-200 HR size exclusion column and eluted over 140 min at 1 ml per minute with 20 mM Tris and 300 ml NaCl. Fractions were tested for DepB activity and the most active fractions were pooled and concentrated by ultrafiltration as above.

### Protein Sequencing

The concentrated pooled sample of the most active fractions was sent for protein sequencing to the Advanced Analysis Centre at the University of Guelph (Guelph, ON, Canada). The results can be found in **Supplementary Table S1**.

### Gene Synthesis and DNA Manipulation

Six potential candidates responsible for the transformation of 3-keto DON to 3-*epi*-DON activity were selected from **Supplementary Table S1**, their subcellular localization was predicted using PSORTb 3.0 (Yu et al., 2010) and SignalP 4.0 (Petersen et al., 2011); all proteins were predicted to be cytosolic. The genes were then codon optimized for synthesis in *E. coli*, synthesized and cloned into pET28A by Genscript (Piscataway, NJ, United States). The genes were inserted to pET28a using NdeI and BamHI restriction sites to produce proteins with N-terminal polyhistidine tags. Their protein sequences can be found in **Supplementary Table S2**. Each construct was transformed separately into *E. coli* BL21; a single colony was selected for protein expression.

### Expression and Purification of Recombinant Proteins

*E. coli* BL21 containing each synthesized construct (see above) was propagated at 37°C in 500 ml of LB medium supplemented with 34 mg l^-1^ kanamycin while shaking at 175 rpm. When the cultures reached an optical density of approximately 0.7, 150 μM of isopropyl β-d-1-thiogalactopyranoside (IPTG) was added to initiate protein expression. The cultures were incubated overnight at 18°C and harvested by centrifugation at 8,000 × *g* for 10 min and frozen at -20°C. Each pellet was thawed and resuspended in 5 ml of 50 mM Tris, pH 8.0, 150 mM NaCl. The cells were then lysed by sonication using the following parameters: 30% amplitude with a cycle of 30 s on, 40 s off for 8 cycles. Lysates were centrifuged at 7,142 × *g* for 30 min to remove the insoluble pellet and filtered through a 0.45 μm filter. The clarified lysates (approximately 5 mL) were then mixed with an equal volume of 50 mM sodium phosphate with 300 mM NaCl and 20 mM imidazole, pH 8.0 (wash buffer) and incubated with 300 μL of HisPure Ni-NTA resin (Thermo Fisher) for 1 h. Each mixture was then briefly centrifuged and the supernatant was removed from the tubes. The beads were resuspended in 1 ml of wash buffer and transferred to 1.5 ml tubes. The tubes were briefly spun, supernatant removed and resuspended with 1 ml of wash buffer. This was repeated for a total of 5 washes. The proteins which bound to the beads were eluted with 1 ml of 50 mM sodium phosphate with 300 mM NaCl and 250 mM imidazole (elution buffer), pH 8.0 and the beads removed by centrifugation. Each of the six samples was then tested for DepB activity.

### Testing the Candidates for Activity

Twenty microliter of purified protein from each construct was added to a reaction containing 400μM NADPH and 100μg ml^-1^ 3-keto-DON in 50 mM Tris, pH 8.0. The reduction in absorbance at 340 nm was monitored using an Ultrospec 3100 *pro* UV/Vis Spectrophotometer (GE Healthcare/Amersham Biosciences). Activity was later confirmed by HPLC analysis as described below.

### HPLC Analysis

Samples and standards (20μL) were separated by a Proteo Phenomenex column (Jupiter, 4 μm, 250 mm × 4.6 mm) attached to a Shimadzu uHPLC system (Mandel Scientific Company, Guelph, ON, Canada). The mobile phases used were: solvent A (100% water) and solvent B (100% acetonitrile). To separate and detect the level of DON and 3-*epi*-DON in a sample the following elution was used: An 11 min isocratic elution consisting of 12% solvent B was used to detect DON and 3-*epi*-DON followed by a column wash of 80% solvent B for 3 min and a re-equilibration of the column at 12% solvent B for 6 min. 3-keto-DON was eluted using the following settings: A 15 min isocratic elution consisting of 23% solvent B was used to detect 3-keto-DON followed by a column wash of 90% solvent B for 3 min and a re-equilibration of the column at 23% solvent B for 6 min. DON, 3-keto-DON and 3-*epi*-DON were detected by monitoring the absorbance at 218 nm and were quantified by comparison to a known standard.

### Purification of DepB

Once a specific protein was identified as DepB a different method for purification was used. The expression was the same except two, 1 L cultures were grown at a time and the pellets pooled. Cells were lysed under the same conditions except 15 sonication cycles were used. The clarified lysate was mixed with an equal volume of wash buffer and incubated with one ml of Ni-NTA agarose for an hour at 4°C. The mixture was then poured into a Flex-Colum (Kimble Chase Life Science, Vineland, NJ, United States) and washed with 40 ml of wash buffer. DepB was eluted with eight ml of elution buffer. The buffer was changed by repeated dilutions in the stirred cell to 50 mM Tris, 150 mM NaCl, pH 8.0. The purified enzyme was then aliquoted stored at -80°C.

### Buffer, pH, and Cofactor Specificity

Reactions assessing the buffer specificity of DepB contained 50 mM of various buffers, 100μg ml^-1^ 3-keto-DON, 400μM NADPH, and 7μg DepB. Each reaction was allowed to proceed for 10 min before it was stopped with acidified methanol. The activity of DepB at various pH values was examined. Reactions contained 50 mM of three component buffer (0.1 M Tris, 0.05 M acetic acid, and 0.05 M 2-(N-morpholino)ethanesulfonic acid) at various pH’s, 100μg ml^-1^ 3-keto-DON, 400μM NADPH, 4.7μg DepB and the reaction was allowed to proceed for 15 min before it was stopped with acidified methanol. Cofactor specificity was tested under the following conditions: reactions contain 50 mM of Tris pH 7.5, 100μg ml^-1^ 3-keto-DON or DON, 400μM NADPH or NADH, NADP or NAD, 4.7μg DepB and the reaction was allowed to proceed for 15 min or overnight before it was stopped with acidified methanol. Reactions were carried out in triplicate and the amount of 3-*epi*-DON produced was assessed by HPLC.

### Thermostability and the Effect of Lyophilization on DepB

The stability of DepB (purified from *E. coli*) and the ability of the enzyme to catalyze the reaction was tested at multiple temperatures. Aliquots of DepB were incubated at a specific temperature between 30 and 70°C for 1 h, and cooled on ice. Heat treated DepB and untreated DepB (kept at 4C) were tested for activity at room temperature (23 ± 2°C). The activity of DepB was monitored from 20 to 60°C in 5°C intervals. Each reaction contained 100μg ml^-1^ 3-keto-DON, 400μM NADPH, 5μg DepB in 50 mM of Tris pH 7.5. The reactions were carried out in triplicate and were allowed to proceed for 7.5 and 15 min before they were stopped by addition of acidified acetonitrile. The amount of 3-*epi*-DON produced per minute was determined by HPLC. Separately, frozen aliquots of DepB were lyophilized, stored at room temperature for 1 week before resuspension in water. Samples which were lyophilized were assessed for activity at 25°C and were compared to those which had been freshly removed from storage at -80°C and thawed on ice. Each reaction contained 100μg ml^-1^ 3-keto-DON, 400μM NADPH, 4.7μg DepB in 50 mM of Tris pH 7.5. The reactions were allowed to proceed for 12 min before they were stopped by addition of acidified methanol. Reactions were carried out in triplicate and the amount of 3-*epi*-DON produced was assessed by HPLC.

### Comparative Genomic Analysis

The amino acid sequences of all predicted CDSs from 19 strains of *Devosia* (*Devosia chinhatensis* GCA_000969445.1; *Devosia epidermidihirudinis* GCA_000971295.1; *Devosia geojensis* GCA_000969415.1; *Devosia insulae* DS-56 GCA_000970465.2; *Devosia limi* DSM 17137 GCA_000970435.1; *Devosia psychrophila* GCA_000971275.1; *Devosia riboflavina* GCA_000743575.1; *Devosia soli* GCA_000970455.1; *Devosia* sp. A16 GCA_001402915.1; *Devosia* sp. DBB001 GCA_000689495.1; *Devosia* sp. H5989 GCA_001185205.1; *Devosia* sp. LC5 GCA_000735585.1; *Devosia* sp. Leaf420 GCA_001425445.1; *Devosia* sp. Leaf64 GCA_001421945.1; *Devosia* sp. Root105 GCA_001424865.1; *Devosia* sp. Root413D1 GCA_001425235.1; *Devosia* sp. Root436 GCA_001426345.1; *Devosia* sp. Root635 GCA_001427605.1; Devosia sp. Root685 GCA_001427875.1) were aligned against the *D. mutans* (*Devosia* sp. 17-2-E-8 GCA_000743515.1) genome sequence with tblastn, using an *e*-value cut-off of 1e-5. The best-scoring alignments from each CDS were visualized with Circos v.0.69.

### Statistical Analysis

Statistical Analyses were performed using GraphPad Prism 7.02 (Muzyka, 2016). All reactions were run in triplicates and where appropriate Tukey’s multiple comparison tests were used to determine if there was a significant difference between treatments.

## Results

### Partial Purification of DepB From *D. mutans* 17-2-E-8 Crude Lysate

After ammonium sulfate precipitation at 30, 50, and 70% of saturation, the fraction containing proteins which salted out between 30 and 50% of saturating ammonium sulfate had the highest level of DepB activity. After dialysis, proteins which did not precipitate during heat treatment were subjected to anion exchange chromatography (**Supplementary Figure S1**). The most active fractions were pooled, concentrated and DepB was further purified by size exclusion chromatography (**Supplementary Figure S2**). The DepB activity of fractions roughly correlated with the intensity of a band at 38 kDa. The most active fractions were pooled, concentrated and used for protein sequencing.

### Identification of DepB

The list of proteins in the sample was quite extensive (**Supplementary Table S1**), with 101 proteins identified based on sequence identity of peptides with the draft *D. mutans* genome (Genbank JQGB00000000). From this list, six candidates were selected based on their predicted function and size (**Supplementary Table S2**). The codon optimized candidate genes were cloned into pET28a and expressed in *E. coli* BL21. After an initial Ni-NTA purification each candidate was tested for DepB activity by measuring the reduction of 3-keto-DON. One gene, fig| 6666666.163324.CDS.25 (GenBank: KFL28068.1), hereafter termed *depB*, was shown to encode a functional protein, DepB. The activity of DepB was confirmed by HPLC, whereby after the addition of DepB to solution 3-keto-DON disappeared from solution while 3-*epi*-DON appeared. The identity of 3-*epi*-DON was confirmed by LCMS/MS. DepB was then re-purified (**Supplementary Figure S3**) and aliquots were stored at -80°C.

### Genomic Location of depB

**FIGURE 2:**
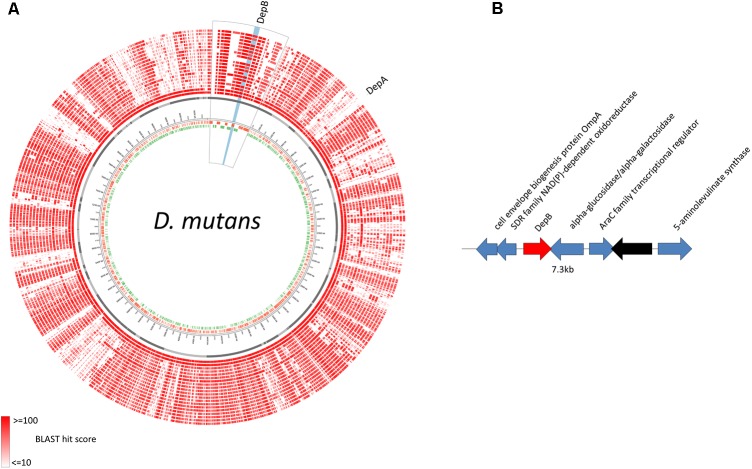
Genomic location of *D. mutans depB* region. **(A)** *D. mutans* genome atlas showing protein sequence similarities with 19 other Devosia strains. The inner three rings represent the forward CDS (green), reverse CDS (red) and contigs (gray). The remaining rings (red) represent the tblastn hits of the predicted proteins from 19 *Devosia* strains against *D. mutans* (inner to outer): *D. mutans*, *Devosia* sp. DBB001, *Devosia* sp. A16, *D. chinhatensis*, *D. epidermidihirudinis*, *D. geojensis*, *Devosia* sp. H5989, *D. insulae*, *Devosia* sp. LC5, *Devosia* sp. Leaf420, *Devosia* sp. Leaf64, *D. limi*, *D. psychrophila, D. riboflavina, Devosia* sp. Root105, *Devosia* sp. Root413D1, *Devosia* sp. Root436, *Devosia* sp. Root635, *Devosia* sp. Root685, *D. soli*. The region surrounding *depB* is magnified (outlined in black) and *depB* highlighted in blue. **(B)** Gene diagram of *depB* (red) and surrounding genes (blue, predicted function; black, hypothetical protein).

The predicted DepB CDS is located on an ∼127 kb contig that is distinct from the ∼128 kb contig on which *depA* is located (**Figure 2A**). These two proteins are therefore not part of the same operon, and are in fact quite distant from one another on the *D. mutans* genome. BLAST comparisons of the *depB* region with 19 other *Devosia* strains demonstrates that the CDSs in this region are mostly conserved among different strains (**Figure 7A**), although synteny is not (data not shown). In *D. mutans*, *depB* is flanked by a predicted alpha-glucosidase/alpha-galactosidase and a SDR family NAD(P)-dependent oxidoreductase; however, both flanking genes are in the opposite orientation of *depB*, indicating that they are not co-transcribed with *depB* (**Figure 2B**).

### Preliminary Biochemical Characterization

**FIGURE 3:**
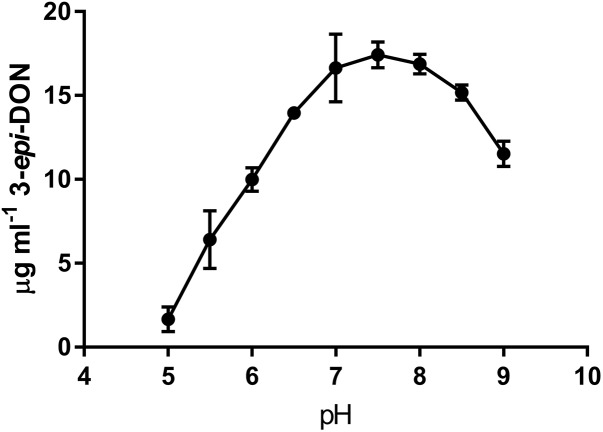
The pH dependency of DepB. Reactions contained 50 mM of three component buffer at various pH-values, 100μg ml^-1^ 3-keto-DON, 400μM NADPH, 4.7μg DepB, the reaction was stopped after 15 min.

The activity of DepB was tested with multiple common buffers. There was no significant difference in the relative activity of DepB between any of the buffers except for ammonium bicarbonate, which was lower than the HEPES buffer (**Supplementary Figure S4**). Tris was ultimately utilized as the buffer in which future experiments were performed. The activity of DepB at pH values from 5.0 to 9.0 was assessed (**Figure 3**). The highest activity of DepB was at a pH of 7.5 but it was not significantly different from activity at near neutral pH. This shows that DepB has a relatively broad pH range in which it can function to transform 3-keto-DON to 3-*epi*-DON.

**FIGURE 4:**
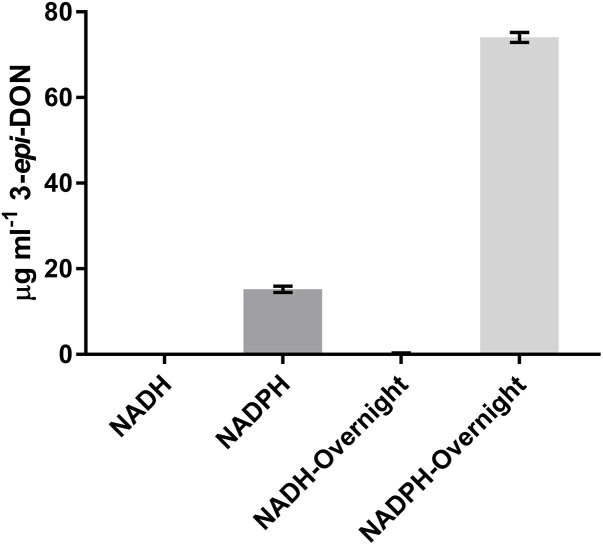
DepB activity using different nicotinamide cofactors. Reactions contain 50 mM of Tris pH 7.5, 100μg ml^-1^ 3-keto-DON, 400μM NADPH or NADH, 4.7μg DepB and the reaction was allowed to proceed for 15 min or overnight before it was stopped with acidified methanol.

It was previously shown that the reduction of 3-keto-DON to 3-*epi*-DON was enhanced by the presence of NADPH (Hassan et al., 2017), so the ability of NADH to be utilized as a cofactor was also tested. After a 15 min incubation with 3-keto-DON, only reactions containing NADPH produced substantial amounts of 3-*epi*-DON, although a small amount of 3-*epi*-DON was also detected after the NADH reaction was left overnight (approximately 16 h) (**Figure 4**). This demonstrates that DepB is indeed NADPH dependent. The ability of DepB to convert DON to 3-keto-DON was tested using NAD^+^ and NADP^+^ as cofactors, but no trace amount of 3-keto-DON could be detected even after the reactions were incubated overnight (data not shown).

**FIGURE 5:**
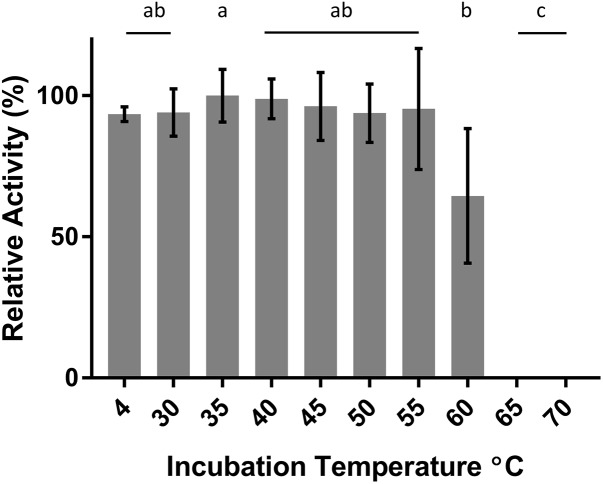
The effect of heat treatment on the activity of DepB. Each reaction contained 100μg ml^-1^ 3-keto-DON, 400μM NADPH, 5μg DepB in 50 mM of Tris pH 7.5. Treated samples were subjected to a 1 h incubation at temperature then cooled on ice. The activity of the enzyme after treatment was plotted relative to the activity to the treatment at 35°C. a, not significantly different from 35°C. b, not significantly different from 60°C. c, not significantly different from 65°C.

**FIGURE 6:**
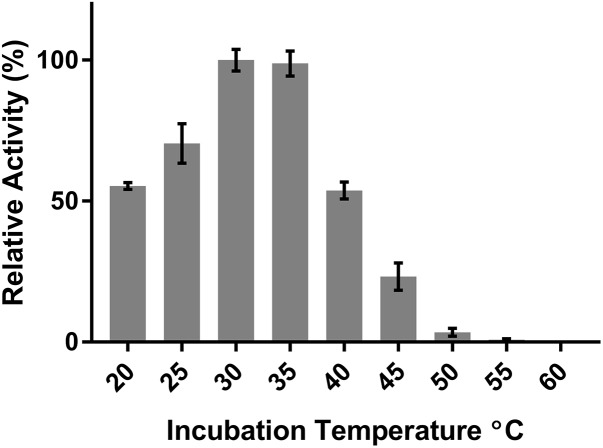
The effect of temperature on the activity of DepB. Each reaction contained 100μg ml^-1^ 3-keto-DON, 400μM NADPH, 5μg DepB in 50 mM of Tris pH 7.5. The activity of the enzyme at each temperature was plotted relative to the activity at 30°C.

**FIGURE 7:**
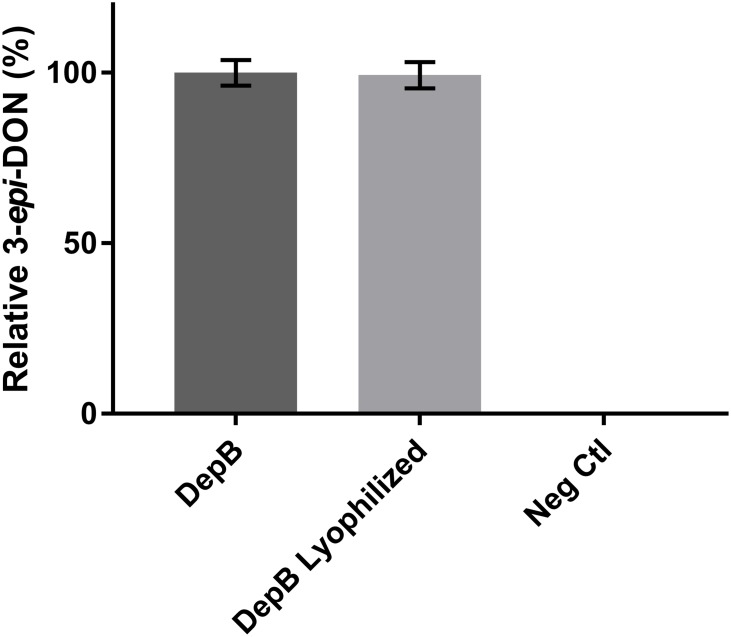
The effect of lyophilization on the activity of DepB. Each reaction contained 100μg ml^-1^ 3-keto-DON, 400μM NADPH, 4.7μg DepB in 50 mM of Tris pH 7.5 and was stopped after 12 min. Activity is relative to DepB which was not lyophilized. Neg Ctl had no enzyme added to the solution.

DepB remained stable when incubated at temperatures from 30 to 55°C (**Figure 5**). The activity was reduced after an incubation at 60°C and abolished after incubations of 65°C and higher. DepB catalyzed the reaction the fastest at 30 and 35°C while had no detectable activity at 60°C (**Figure 6**). As this enzyme may have commercial uses, its activity after being lyophilized and resuspended in water was assessed. There was no significant change in the activity of DepB after lyophilization (**Figure 7**).

## Discussion

Here we have identified the second enzyme in the Dep pathway, DepB, responsible for the reduction of 3-keto-DON to 3-*epi*-DON. DepB is the first enzyme identified that is capable of performing this important detoxification function and its identification may be a key component of developing a system to mitigate the impact of DON in grains. The identification of DepB from a partially purified lysate from its native host shows that it is unnecessary to completely purify enzymes in order to identify an enzyme of interest. A combination of ammonium sulfate precipitation, mild heat treatment, followed by two column chromatography steps did not drastically reduce the number of proteins in the sample but increased the relative abundance of the protein of interest to a degree that made the identification of the enzyme possible. In essence, this work demonstrates that a partial purification combined with biochemical knowledge about the enzyme(s) involved (such as cofactor utilization or molecular size) is sufficient to generate a short list of candidate genes. Having these genes synthesized and codon optimized can aid in rapid screening of the candidate genes and identification of the gene of interest.

The gene identified as *depB* in the presented work is annotated by the Rapid Annotation using Subsystem Technology (RAST) server as a putative oxidoreductase and in the National Center for Biotechnology Information (NCBI) as an aldo/keto reductase or dehydratase (as were all of its closest BLAST hits). DepB is a member of the AKR family, containing the signature TIM barrel motif and the predicted catalytic tetrad (D48, Y53, K81, and H122). Although *depB* and its surrounding gene sequences are largely conserved among different *Devosia* strains, in most cases little to no synteny exists in this region. Together with the fact that *depB* is in the opposite orientation to its surrounding genes in *D. mutans*, it is difficult to infer its original function based on genomic context. Notably, *depB* is located distantly to *depA* in the *D. mutans* genome, despite the two proteins participating in a common biochemical pathway. This suggests that the two genes were not initially acquired as a single functional unit with DON transforming activity (e.g., via horizontal gene transfer), but rather had different initial functions that eventually co-evolved to metabolize DON. It is also interesting to note that *depB* was annotated as “putative” by the RAST server and has general annotation by NCBI (Aziz et al., 2008). Genes with a less specific annotation may be more suitable candidates when investigating novel functions as those with more specific annotations may be quite similar to known proteins, thus lacking novel functions. Although this may not always be true, it may prove helpful when only a limited number of candidates can be selected.

As DON is one of the most economically important mycotoxins worldwide, identifying detoxification mechanisms has potentially major implications for the agriculture industry (Ji et al., 2016). Although many microorganisms which can detoxify DON have been known for some time, due to the demanding conditions of their growth and more importantly to issues related to their safe usage in feed and food matrixes, they are not currently used in practical applications under industrial settings. Significant efforts have been put into identifying enzymes capable of detoxifying DON, but until recently, have been largely unsuccessful (Karlovsky, 2011; Ji et al., 2016). Now that the enzymes responsible for Dep have been identified, purified enzymes could be used to detoxify DON or, the enzymes could be incorporated into microorganisms (such as yeasts or lactic acid bacteria) which are currently part of production processes.

As DepB may be useful in the future as part of a system to detoxify DON, its ability to work in various buffers and at a range of pH was tested. DepB effectively transformed 3-keto-DON to 3-*epi*-DON in all the buffers tested. This is important as the enzyme may be utilized in very different matrices and may need to be utilized in the presence of other enzymes requiring specific conditions. DepB also catalyzes the reaction reasonably well over a broad pH range (5–9) which is important as certain uses might have a lower pH (cereal milling) while others are closer to neutral pH (alcohol fermentation). The ability of DON detoxifying enzymes to work under multiple processing conditions will be advantageous (and might be necessary in some cases) for the development of an effective detoxification system.

DepB is clearly a NADPH dependent enzyme. Although catalysis is possible with NADH, it is approximately 5,000-fold less efficient. Previously, an enzyme from *Sphingomonas* sp. strain S3-4, AKR18A1 (GenBank: MF314460.1), with moderately high sequence similarity (42%) to DepB was shown to convert DON to 3-keto-DON, using NADP as a cofactor (He et al., 2017). DepB was unable to convert DON to 3-keto-DON even after prolonged incubation. This enzyme was identified in *Sphingomonas* sp. strain S3-4, a strain capable of transforming DON to 3-keto-DON and 3-*epi*-DON in sequence. While unable to complete this reaction, DepB is able to complete the second step of the pathway, transforming 3-keto-DON to 3-*epi*-DON. It is interesting that in the DON detoxification pathway from *D. mutans* 17-2-E-8 the first step is completed by a PQQ-dependent dehydrogenase while the second set is performed by a NADPH-dependent aldo-keto reductase (Carere et al., 2017) whereas in *Sphingomonas* sp. strain S3-4, the first step is performed by an aldo-keto reductase that shares the sequence homology of the second step enzymes in the 17-2-E-8 strain (He et al., 2017). Considering both active sites bind the same structure with the exception of the 3′ group, small changes in the active site might allow for the altered stereospecificity. Comparing the involved active sites and possibly identifying the responsible residues that dictate outcomes of such enzyme/substrate interactions merits further investigation.

A 1 h heat treatment at 55°C (or lower) did not significantly affect the activity of DepB, a treatment at 60°C did reduce activity while treatments at 65°C or higher abolished activity. Stability at moderately high temperatures may be critical if DepB is to be used in certain industries (such as corn milling) as several processing steps occur at elevated temperatures. Although DepB is stable at 55°C, it had drastically reduced activity at this temperature compared to room temperature. DepB has maximal activity between 30 and 35°C, while retaining robust activity between 20 and 40°C. DepB catalyzes the conversion of 3-keto-DON to 3-*epi*-DON slowly at temperature at or above 50°C, however, it could retain activity until the temperature becomes lower. If this enzyme is to be incorporated into a system to detoxify DON in corn milling, stability at 55°C could be a significant advantage over less stable enzymes. A promising property of DepB is that it was able to be lyophilized, and resuspended in water without a loss of activity. This could be quite important and a valued characteristic if a product were to be developed as it would enable the product to be more easily shipped and stored by the end users, especially if those users were to be farmers treating feed.

The identification of DepB, the second enzyme in the Dep pathway provides an opportunity for developing a system to detoxify DON in contaminated grains. Currently many DON mitigation strategies focus on pre-harvest prevention of *Fusarium* sp. infection or prevention of fungal growth post-harvest (Kabak et al., 2006). The Dep pathway may lead to a viable route to detoxify DON, thus reducing/eliminating the negative aspects of DON toward livestock. There are several possibilities for both the delivery method and applications of the system. Purified enzymes could be utilized during the corn milling process, during ethanol fermentation process or direct to animal feed. Alternatively, microorganisms expressing the system could be incorporated into pre-existing systems. In addition, if the system was expressed in wheat, it may be able to alleviate the toxicity of DON to the plant, thus reducing losses due to *Fusarium*. Although both enzymes in the Dep pathway have now been identified, significant work is still needed in order to develop these enzymes into a system capable of having a meaningful impact to industry.

## Author Contributions

TZ, YH, and JC, conceived the project. JC and YH designed and carried out the experiments. DL provided the genetic analysis. JC prepared the manuscript and JC, YH, DL, and TZ contributed to the final version of the paper. All authors read and approved the final manuscript.

## Conflict of Interest Statement

The authors declare that the research was conducted in the absence of any commercial or financial relationships that could be construed as a potential conflict of interest.

## Supplementary Material

FIGURE S1 **(A)** Elution profile of the protein solution separated by anion exchange. Fractions with the highest activity are marked (^∗^) and were pooled for further purification. **(B)** SDS–PAGE of fractions 8-13. Lane 1, protein standards, Lanes 2-3, Fractions from DepA purification, Lane 4, Flow Through, Lanes 5-10, Fractions 8-13; respectively.

FIGURE S2 **(A)** Elution profile of the protein solution separated by size exclusion. Most active fractions indicated with a (^∗^) **(B)** SDS–PAGE of fractions 10-18. Lane 1, protein standards, Lane 2-9 Fractions 10-18; respectively.

FIGURE S3 Purified DepB from *E. coli*. Lane 1, protein standards. Lane 2, crude lysate. Lane 3 flow through. Lane 4, final wash. Lane 5, purified DepB.

FIGURE S4 The relative activity of DepB with various buffers. Each reaction contained 50 mM of the respective buffer, 100 μg ml^-1^ 3-keto-DON, 400 μM NADPH, 7 μg DepB and the reaction was allowed to proceed for 10 min before it was stopped with acidified methanol. Activity is relative to the activity in HEPES. ^∗^Activity in ammonium bicarbonate was significantly lower than in HEPES as determined by a Tukey’s multiple comparison test.

TABLE S1 Protein sequencing results.

TABLE S2 Candidate genes for having DepB activity.
