# Supplementary figures and images for: The Identification of DepB: An Enzyme Responsible for the Final Detoxification Step in the Deoxynivalenol Epimerization Pathway in Devosia mutans 17-2-E-8
Source: Front Microbiol. 2018 Jul 17;9:1573. doi: 10.3389/fmicb.2018.01573 (PMC6056672; doi:10.3389/fmicb.2018.01573)

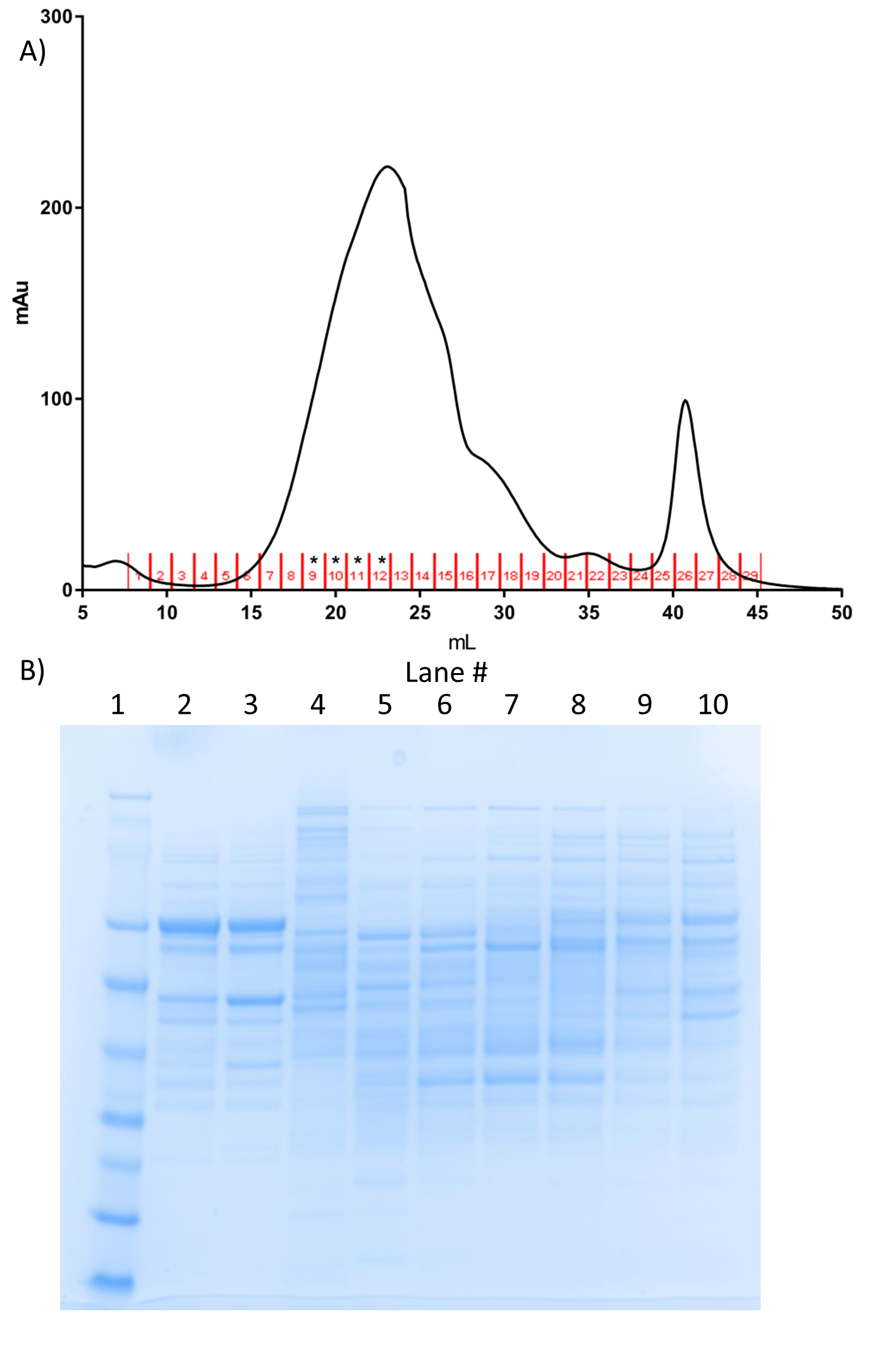

Supplement: FIGURE S1 — (A) Elution profile of the protein solution separated by anion exchange. Fractions with the highest activity are marked (∗) and were pooled for further purification. (B) SDS–PAGE of fractions 8-13. Lane 1, protein standards, Lanes 2-3, Fractions from DepA purification, Lane 4, Flow Through, Lanes 5-10, Fractions 8-13; respectively. [file Image_1.TIF]

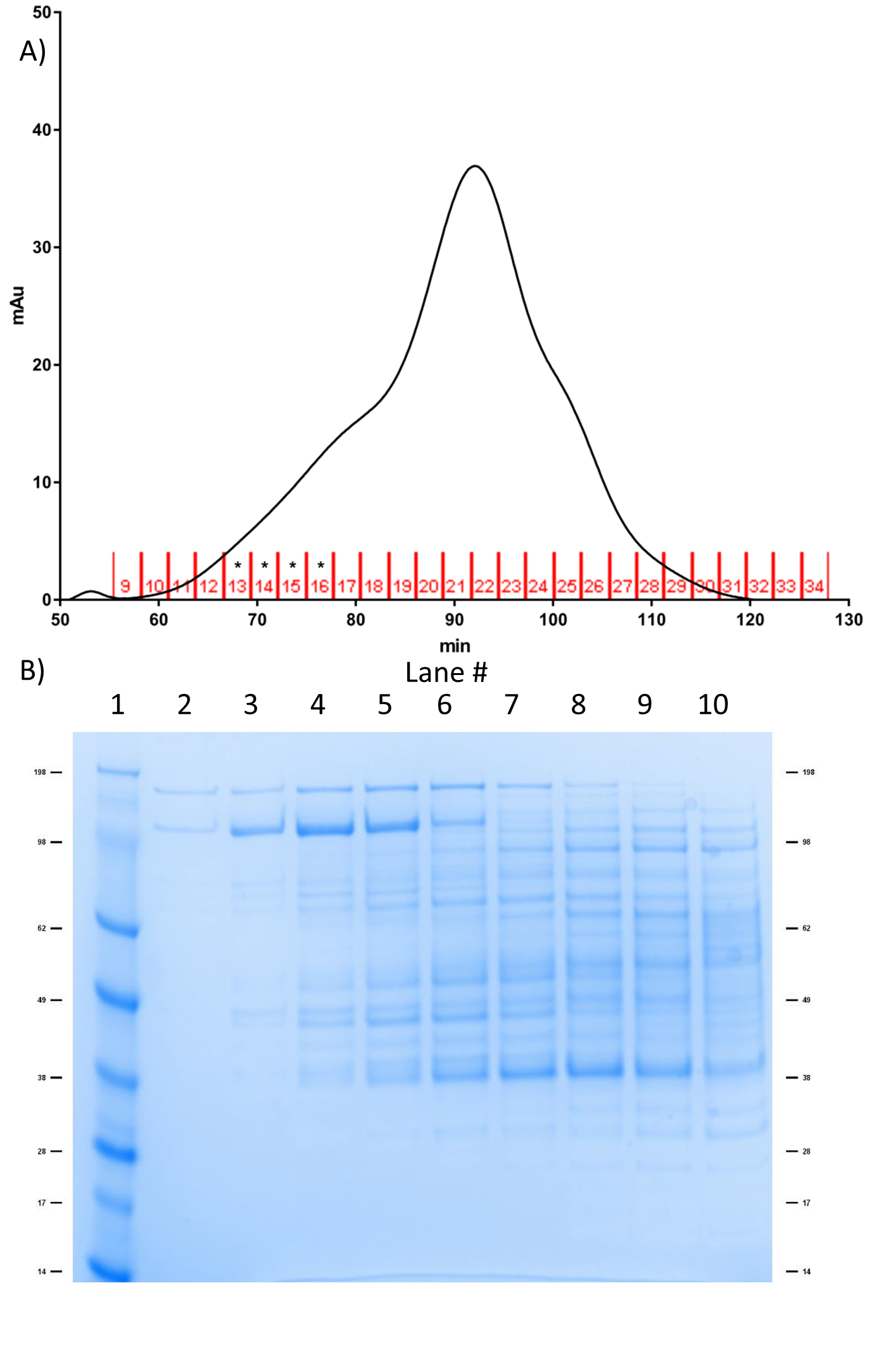

Supplement: FIGURE S2 — (A) Elution profile of the protein solution separated by size exclusion. Most active fractions indicated with a (∗) (B) SDS–PAGE of fractions 10-18. Lane 1, protein standards, Lane 2-9 Fractions 10-18; respectively. [file Image_2.TIF]

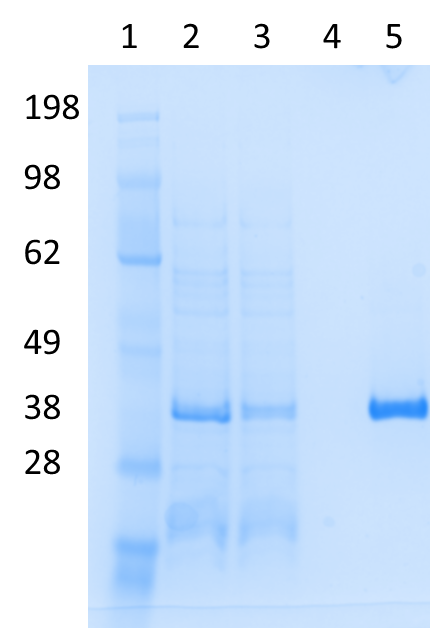

Supplement: FIGURE S3 — Purified DepB from E. coli. Lane 1, protein standards. Lane 2, crude lysate. Lane 3 flow through. Lane 4, final wash. Lane 5, purified DepB. [file Image_3.TIF]

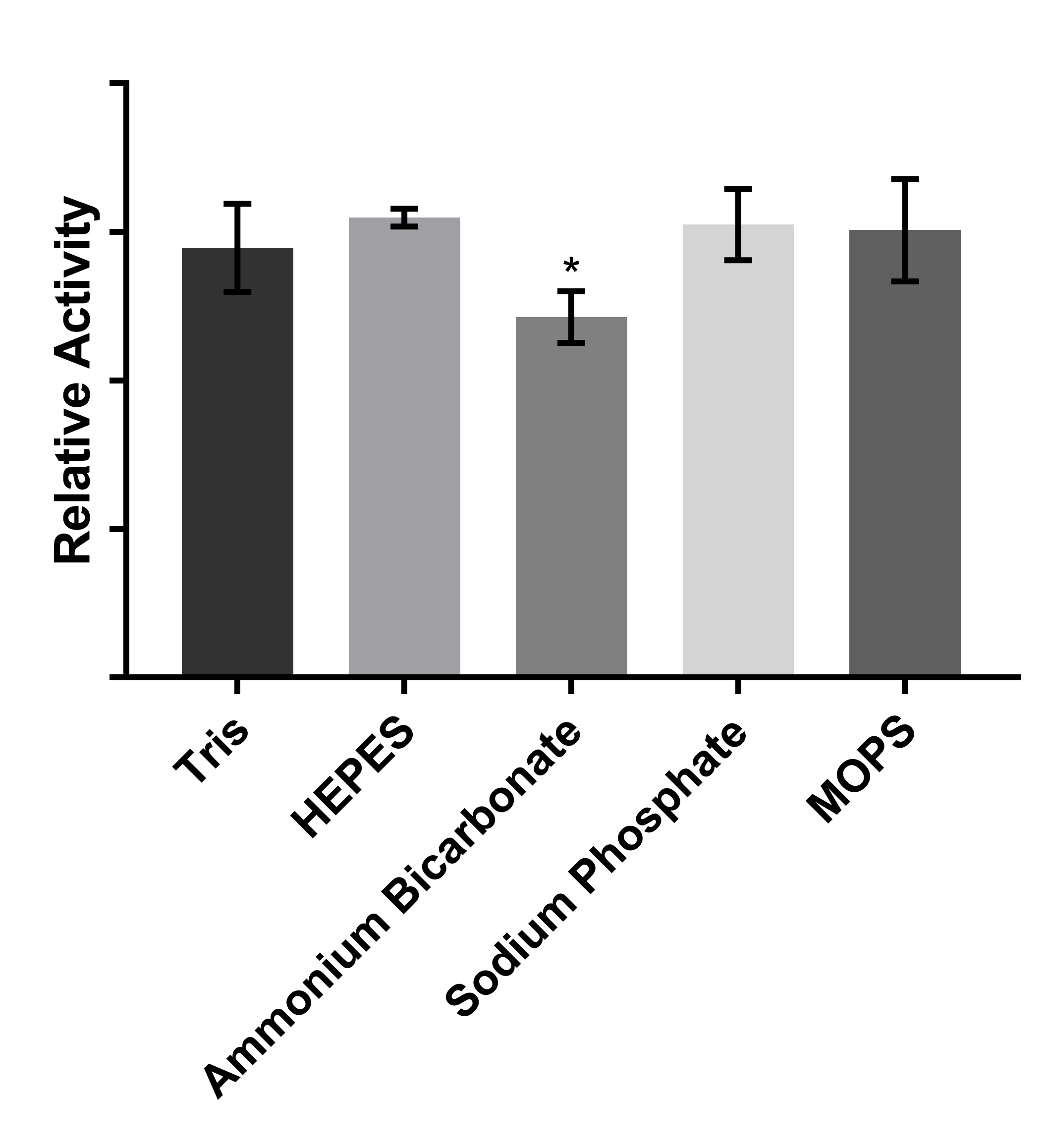

Supplement: FIGURE S4 — The relative activity of DepB with various buffers. Each reaction contained 50 mM of the respective buffer, 100 μg ml-1 3-keto-DON, 400 μM NADPH, 7 μg DepB and the reaction was allowed to proceed for 10 min before it was stopped with acidified methanol. Activity is relative to the activity in HEPES. ∗Activity in ammonium bicarbonate was significantly lower than in HEPES as determined by a Tukey’s multiple comparison test. [file Image_4.TIF]
